# Predictor of Surgical Failure Following Transvaginal Mesh Repair Using Minimally Invasive Prolapse System Device (MIPS)

**DOI:** 10.3390/jcm13185352

**Published:** 2024-09-10

**Authors:** Yao-Yu Yang, Zi-Xi Loo, Kun-Ling Lin, Cheng-Yu Long

**Affiliations:** 1 Department of Obstetrics and Gynecology, Kaohsiung Medical University Hospital, Kaohsiung Medical University, Kaohsiung 80756, Taiwan; 1120307@kmuh.org.tw (Y.-Y.Y.); 1030394@kmuh.org.tw (Z.-X.L.); 960233@kmuh.org.tw (K.-L.L.); 2 Department of Obstetrics and Gynecology, Kaohsiung Municipal Ta-Tung Hospital, Kaohsiung Medical University, Kaohsiung 80145, Taiwan; 3 Department of Obstetrics and Gynecology, College of Medicine, Kaohsiung Medical University, Kaohsiung 80756, Taiwan; 4 Department of Obstetrics and Gynecology, Kaohsiung Municipal Siaogang Hospital, Kaohsiung Medical University, Kaohsiung 81267, Taiwan

**Keywords:** pelvic organ prolapse, recurrence, surgical failure, transvaginal mesh repair, uterine prolapse

## Abstract

**Background:** The Minimally Invasive Prolapse System (MIPS) device, a novel single-incision transvaginal mesh, represents recent advancements in mesh technology, providing lightweight, biocompatible support for pelvic organ prolapse while reducing erosion, allowing for customization and improving surgical outcomes. This study aimed to identify factors associated with pelvic organ prolapse (POP) recurrence after transvaginal mesh (TVM) repair using the Minimally Invasive Prolapse System device. **Methods:** Two hundred and eighteen women with symptomatic stage II to IV POP underwent TVM. Preoperative and postoperative assessments included urinalyses and pelvic examinations using the POP quantification (POP-Q) staging system. **Results:** During a follow-up period of 12–46 months, 7 of 218 (3.2%) women experienced POP recurrence. Univariate analysis was conducted to identify predictors of surgical failure, revealing no significant differences in body mass index, POP stage, or preoperative urinary symptoms between the recurrence and success groups (*p* > 0.05). However, functional urethral length <20 mm based on urodynamics (*p* = 0.011), ICI-Q scores ≥7 (*p* = 0.012), and the first 60 surgical cases (*p* = 0.018) were significant predictors of surgical failure. Multivariate logistic regression confirmed these findings. **Conclusions:** Functional urethral length <20 mm, ICI-Q scores ≥7, and limited surgical experience were significant predictors of TVM failure using the Minimally Invasive Prolapse System kit. POP recurrence after mesh repair is less likely beyond the learning curve.

## 1. Introduction

Pelvic organ prolapse (POP) is a significant issue in gynecology, impacting up to 40% of women who have given birth [1]. Its prevalence escalates with age and parity, imposing considerable limitations on quality of life due to the resultant dysfunctions. The lifetime risk of requiring surgical intervention for POP ranges between 7% and 19%, with those having undergone multiple procedures facing a greater than 50% likelihood of additional surgeries [2,3,4,5].

In response to mesh-related complications highlighted by the FDA in 2008, stringent regulations were introduced by 2016, culminating in a ban on transvaginal mesh for POP surgery in 2019 [6]. Despite the FDA’s stance, global practices vary significantly. In some regions, vaginal mesh remains a standard treatment, while others adopt a more cautious approach. Following the 2015 evaluation by the Scientific Committee on Emerging and Newly Identified Health Risks (SCENIHR), the European Union endorses vaginal mesh for recurrent or high-risk POP. It advises certified surgeons and emphasizes meticulous patient selection and counseling. The German guidelines, supported by the German Working Group of the Scientific Medical Societies (AWMF), the European Urogynecological Association (EUGA), and the European Association of Urology (EAU), authorize the selective use of transvaginal meshes for POP repair, claiming they are more successful and have lower recurrence rates compared to native tissue repair.

Advances in mesh technology have led to the development of lighter, macroporous, and isoelastic meshes, reducing erosion rates. The Minimally Invasive Prolapse System mesh (MIPS) (DIMA S.L., Inc., Calatayud, Zaragoza, Spain) is a biocompatible, ultra-lightweight polypropylene mesh (28 gr/m^2^) used for treating apical prolapse involving the anterior or posterior compartment. This mesh is inserted transvaginally through a single incision using the disposable instruments included in the mesh kit. The anchoring fixation mechanism of the Minimally Invasive Prolapse System device for sacrospinous fixation, crafted from 100% biocompatible peek material, ensures maximum anchoring strength while maintaining comfort and facilitating rapid fixation. It prevents shrinkage and promotes the maintenance of vaginal length post-procedure. The system also allows customization of graft placement and adjustability, reducing the risk of wrinkles and minimizing the likelihood of erosion or extrusion.

According to a systematic review and meta-analysis by C-Y Zhang et al. [7], there is no compelling evidence indicating that the anatomical and subjective success rates for sacrocolpopexy are superior to those of transvaginal mesh surgery. Moreover, with comparable success rates, the latest generation of transvaginal mesh benefits from advancements in material and design that reduce complications, while also requiring shorter surgical durations and effectively addressing issues associated with multiple compartment prolapse.

Few studies examine the outcomes, complications, and predictive factors for surgical failure following transvaginal mesh repair with the Minimally Invasive Prolapse System device. Exploring the potential causes behind surgical failures proves to be a compelling area of research. Additionally, the differences between outdated meshes, which are no longer in use, and the newer single-incision TVM continue to pique interest. This study seeks to evaluate these factors and the effectiveness of the Minimally Invasive Prolapse System device in our clinical practice, identifying elements linked to recurrent POP.

## 2. Materials and Methods

### 2.1. Study Design

Between January 2019 and June 2023, our research included 225 consecutive patients diagnosed with POP stage II or greater, according to the POP quantification (POP-Q) staging system [8]. These patients underwent TVM procedures with the Minimally Invasive Prolapse System device at a single medical facility, performed by a single surgeon. Approval for the retrospective data analysis was accepted by the Institutional Review Board (IRB) of our hospital (approval date: 30 January 2019; IRB number: KMUHIRB-E(I)-20190015). 

Participants with uterine disorders were slated for a concurrent hysterectomy, whereas those with an intact uterus were given the option to preserve it. Concomitant mid-urethral slings (MUS) operations with Altis (Coloplast Corp., Minneapolis, MN, USA), one of single-incision slings (SIS), were carried out in patients with either current or occult urodynamic stress incontinence (USI), in the absence of a specific refusal of further surgery. Occult USI was diagnosed preoperatively via a positive cough stress test with prolapse reduction using vaginal packing during cystometry. Due to incomplete medical records, 7 women were excluded from the study, leaving a total of 218 participants for the final analysis.

All participants underwent urinalysis and a pelvic examination utilizing the POP-Q system both preoperatively and postoperatively. Follow-up visits were scheduled at 1, 2, 3, 6, and 12 months after receiving the procedure, and then continued biannually thereafter. Recurrence was characterized as the most dependent part of POP reaching stage II or higher, with the leading edge of the prolapse measuring −1 cm or more. 

Potential risk factors for surgical failure after mesh surgery were examined, including demographics, urinary symptoms, preoperative urodynamic parameters, and subjective satisfaction assessed via questionnaires like the Overactive Bladder Symptom Scores (OABSS) [9], Urogenital Distress Inventory (UDI-6), Incontinence Impact Questionnaire (IIQ-7) [10], International Consultation on Incontinence modular Questionnaire–Short Form (ICIQ-SF) [11], and Pelvic Organ Prolapse Distress Inventory 6 (POPDI-6) [12].

### 2.2. Surgical Technique

The surgical technique for addressing apical and anterior compartment prolapse with the Minimally Invasive Prolapse System device included a bilateral dissection of the anterior vaginal wall up to the arcus tendinous and a digital identification of the sacrospinous ligament. The anchoring applicator was introduced through a vaginal incision and positioned on the sacrospinous ligament roughly 1 to 2 cm medially and posteriorly to the ischial spine. In the course of anterior mesh repair, the superior arms of the device were directed towards the upper medial angle of the obturator foramen, aligning with the urethral orifice. All patients were administered prophylactic antibiotics, specifically 1 g of intravenous cefazolin (Cefamezin, Fujisawa, Tokyo, Japan), prior to surgery. The surgery was conducted under general anesthesia. The uterus was kept intact as long as no abnormalities were detected via ultrasound.

### 2.3. Statistical Analysis

Categorical variables were analyzed using either the chi-square test or Fisher’s exact test. A logistic regression model was utilized to assess the independent predictive value of the variables. Statistical significance was acknowledged when *p* < 0.05. We evaluated the effectiveness of tests in distinguishing surgical outcomes between the groups. While the failure group had a small sample size, we considered several factors, including uterine prolapse rates and surgical experience, to analyze the differences between the two groups. Our findings indicated that the power of discrimination for these two key predictors exceeded 85%.

## 3. Results

The demographic details for the 218 women participating in the study were presented in Table 1. The ages of the patients spanned from 49 to 88 years, with an average age of 68.9 years. Parity varied from 1 to 7, averaging 2.99. Among the participants, 101 (46.33%) were postmenopausal, and 12 (5.5%) had previously undergone a hysterectomy. All participants underwent transvaginal anterior procedures, with 49 (22.5%) also receiving both transvaginal anterior and posterior repair for POP. Concurrent vaginal hysterectomy was carried out in 31 (14.2%) participants, and concomitant cervical amputation was carried out in 28 (12.8%) patients. Additionally, 87 (39.9%) patients required mid-urethral sling procedures to address urinary stress incontinence (USI) (Table 2). After an average follow-up period of 6 months, seven (3.2%) women experienced a recurrence of POP. The overall rate of vaginal erosion in the study was 1.9% (4 out of 218), and all cases occurred in the success group (Table 3).

A univariate analysis of patient demographics and preoperative urodynamic measurements was performed to pinpoint potential risk factors for POP recurrence after TVM surgery, with the findings detailed in Table 3 and Table 4. The analysis revealed no significant variations between the groups concerning age, parity, body mass index, diabetes, hypertension, previous surgeries, POP status, concomitant procedures, and most preoperative urinary symptoms, urodynamic measurements, and satisfaction scores (*p* > 0.05). Nonetheless, surgical experience, functional urethral length, and ICIQ score emerged as three significant indicators of surgical failure. Specifically, the occurrence of POP recurrence was more prevalent in the initial 60 cases (Fisher’s exact test; *p* = 0.018), was higher among women whose functional urethral length was under 20 mm (Fisher’s exact test; *p* = 0.011), and was more frequent among individuals with an ICIQ score of 7 or greater (Fisher’s exact test; *p* = 0.012).

## 4. Discussion

Over the last decade, the utilization of synthetic implants for POP repair has significantly increased despite the U.S. FDA’s ban on TVM, due to the higher failure rates associated with native tissue repairs [13]. However, recommendations from the SCENIHR and joint guidelines from Germany, Austria, and Switzerland support the use of TVM implants for recurrent cases. Additionally, these guidelines suggest considering TVM to be an option for primary interventions in certain scenarios. According to Tailor et al., TVM repair has been found to be as safe as native tissue repair in terms of serious device-related or procedure-related adverse events up to three years post-surgery [14].

Given the critical importance of synthetic mesh, along with the surgeon’s expertise and careful patient selection, improvements in mesh technology, including alterations in material composition, improved designs, and reduced mesh densities, have played a pivotal role in markedly decreasing the frequency of adverse events. The use of advanced lightweight polypropylene mesh in POP surgeries is believed to enhance short-term anatomical outcomes more effectively than traditional natural tissue repairs. Furthermore, it contributes to lowering the recognized risks associated with mesh-related complications [14].

Gert Naumann et al. demonstrated that the Calistar-S mesh (Promedon, S.A., Córdoba, Argentina) was a viable and safe option for mesh-augmented repair of recurrent or complex anterior POP [15]. They noted that for certain individuals, the advantages of utilizing mesh in POP repairs surpassed the associated risks [15]. The Minimally Invasive Prolapse System device, which offers adjustable graft placement and customization, presents a novel alternative for transvaginal anterior repairs. This newly introduced mesh was incorporated into our study to assess its effectiveness, potential complications, and the factors that may contribute to surgical failure.

In this study, the surgical success rate of 96.7% (211 out of 218) corresponds with the results of our prior research, which observed a 96% resolution rate using the Uphold system at the 1-year follow-up [16]. This is consistent with findings from Gert Naumann et al., who reported a 98.0% success rate with the Calistar-S mesh at 6 months post-surgery [15], and Tsia-Shu Lo et al., who observed the same success rate with the Calistar-S mesh at 1 year post-surgery [17].

The amount of preoperative surgical experience significantly impacted the success of TVM surgeries, with the majority of failures (71.4%, five out of seven cases) occurring within the first 60 procedures, indicative of a learning curve. Additionally, all instances of recurrence took place within the first half year post-operation, suggesting a decrease in failure rates as experience accumulated beyond this timeframe. These observations are consistent with our previous study, which underscored the critical role of surgical expertise in the initial 50 cases as a determinant of outcome success in transobturator TVM procedures [18]. Accurate positioning of the mesh around the pericervical ring or vaginal vault is essential in TVM surgeries. Any misplacement during the early learning phases can compromise pelvic support and heighten the likelihood of POP recurrence. The challenge of accurate placement is further exacerbated by the Minimally Invasive Prolapse System device, which utilizes a single-incision approach and does not require needles for positioning the arms near the obturator muscle.

Furthermore, a functional urethral length of less than 20 mm and an ICIQ score of 7 or higher are crucial factors influencing surgical outcomes. The likely reason for this is that more complex conditions are associated with a shorter urethral length and elevated ICIQ scores. Such conditions typically include bladder dysfunction and urinary tract complications, which are common in cases of multi-compartmental POP. Effective treatment requires precise, site-specific repairs of each defect, whether or not a hysterectomy is performed.

Advancements in prosthetic materials have undoubtedly enhanced safety, leading to a minimized impact on the vaginal mucosa and a subsequent reduction in complication rates. In our research, the total vaginal erosion rate was observed at 1.9% (4 out of 218), which is lower than the 2.8% rate found in our earlier studies utilizing the Uphold system [16]. This rate is also considerably lower than the 5.6% erosion rate reported by Gert Naumann et al. for the Caelistar-S mesh [15]. The reduced rate of erosion with the Minimally Invasive Prolapse System device can also be attributed to its adjustable and customizable features, which likely play a role in diminishing erosion risks.

Numerous postmenopausal women frequently experience varying urinary symptoms, with many opting for TVM surgery [19]. Prior research indicates that older age can contribute to failures in TVT-O procedures due to urogenital atrophy, as estrogen and collagen play vital roles in maintaining the integrity of the urethral and anterior vaginal walls [20]. However, in this research, being over 60 and menopausal status did not significantly affect the recurrence of POP, aligning with findings from our more recent studies [18]. Further research is necessary to delve into the distinct causes behind incontinence and POP.

Our latest research indicates that outcomes such as anatomical correction, alleviation of lower urinary tract symptoms, and the incidence of mesh extrusion are similar regardless of whether the uterus is preserved during TVM repair [21]. This study further supports these findings, showing that the success rate of TVM repair is consistent, irrespective of whether a hysterectomy is performed or not.

The strength of this study is rooted in the straightforward and effective surgical methods applied. All participants received an anterior transvaginal mesh repair, and in some instances, multiple interventions were necessary. All surgeries were conducted by the same surgeon at a single tertiary care center, ensuring consistency and reducing variability that might have arisen from different surgical hands or facilities. A limitation of this research, however, was the relatively small sample size within the failure group, likely resulting from the high success rates of the TVM procedures. Furthermore, the duration of follow-up was inadequate for drawing long-term conclusions. There is a need for more extensive studies over longer periods to overcome these shortcomings. Despite these obstacles, our findings reliably represent contemporary clinical practices and offer significant insights into the effectiveness of the Minimally Invasive Prolapse System device within the intended patient population.

## 5. Conclusions

Until now, there are no definitive predictors of POP recurrence after TVM surgery. However, with improvements in material technology and increasing surgical expertise, the incidence of this condition is decreasing. Nevertheless, it remains essential to discuss potential outcomes with patients during the preoperative consultation phase, particularly given the varying global perspectives on the use of TVM. According to our research, a functional urethral length under 20 mm, an ICI-Q score of 7 or higher, and the surgeon’s experience have emerged as significant predictors of surgical failure when using the Minimally Invasive Prolapse System device for TVM. It seems that the likelihood of POP recurrence after TVM surgery diminishes after overcoming the initial learning curve or passing the 6-month postoperative mark. These findings could be invaluable in helping surgeons anticipate and mitigate the risk of recurrence in POP surgeries.

## Figures and Tables

**Table 1:** Demographic characteristics of women (n = 218) with POP following TVM repair.

	n = 218
Age (yrs)	68.91 ± 8.49
Parity	2.99 ± 1.14
BMI (kg/m^2^)	24.37 ± 3.63
Hormone therapy	39 (17.89)
Menopause	101 (46.33)
Hypertension	110 (50.46)
Diabetes mellitus	52 (23.85)
Hysterectomy	12 (5.5)
Previous POP surgery	5 (2.29)
SUI surgery	3 (1.38)
Follow-up (months)	12–46 months

Data are given as mean ± standard deviation or n (%). TVM, transvaginal mesh; BMI, body mass index; POP, pelvic organ prolapse; SUI, stress urinary incontinence.

**Table 2:** Concomitant procedures with Minimally Invasive Prolapse System device in this study (n = 218).

Procedures	n (%)
Cervical amputation	28 (12.8)
Anterior colporrhaphy with mesh insertion	218 (100)
Vaginal hysterectomy	31 (14.2)
Mid-urethral sling insertion	87 (39.9)
Posterior colporrhaphy	49 (22.5)

**Table 3:** Analysis of clinical features in the success and the failure groups.

		Success (n = 211)	Failure (n = 7)	*p* Value	OR
Age (yrs)	<60	26 (12.32)	0 (0)	1	
	≧60	185 (87.68)	7 (100)		
Parity	<4	164 (77.73)	5 (71.43)	0.6559	
	≧4	47 (22.27)	2 (28.57)		
BMI (kg/m^2^)	<25	130 (61.61)	4 (57.14)	1	
	≧25	81 (38.39)	3 (42.86)		
Past history	HT	39 (18.48)	0 (0.00)	0.3567	
	Menopause	99 (46.92)	2 (28.57)	0.4545	
	Hypertension	106 (50.24)	4 (57.14)	1	
	Diabetes mellitus	50 (23.70)	2 (28.57)	0.6726	
	Hysterectomy	12 (5.69)	0 (0.00)	1	
	Previous POP	5 (2.37)	0 (0.00)	1	
	SUI surgery	3 (1.42)	0 (0.00)	1	
Involved compartment	Cystocele	149 (70.62)	3 (42.86)	0.2027	
	Uterine prolapse	91 (43.13)	2 (28.57)	0.7014	
	Vault prolapse	16 (7.58)	2 (28.57)	0.1056	
	Rectocele	12 (5.69)	1 (14.29)	0.3538	
	All	45 (21.33)	3 (42.86)	0.1814	
Concomitant surgery	Cervical amputation	28 (13.27)	0 (0.00)	0.5989	
	VTH	31 (14.69)	0 (0.00)	0.5969	
	MUS insertion	84 (39.81)	3 (42.86)	1	
Mesh erosion		4 (1.90)	0 (0.00)	1	
Pre-op symptoms	Frequency	131 (62.38)	3 (42.86)	0.4319	
	SUI	131 (62.09)	5 (71.43)	0.7134	
	UUI	122 (58.10)	6 (85.71)	0.2443	
	Incomplete emptying	178 (84.76)	6 (85.71)	1	
	Hesitancy	147 (70.00)	5 (71.43)	1	
	Nocturia	168 (79.62)	7 (100.00)	0.3498	
Surgical experience	First 60 case	55 (26.07)	5 (71.43)	0.0181	7.09 (1.34, 37.61)
	61–218 case	156 (73.93)	2 (28.57)		

Data are given as n (%). BMI, body mass index; HT, hormone therapy; POP, pelvic organ prolapse; SUI, stress urinary incontinence; VTH, vaginal hysterectomy; MUS, mid-urethral sling; Pre-op, preoperative; UUI, urge urinary incontinence.

**Table 4:** Comparison of preoperative urodynamic and POP-Q parameters in the success and the failure groups.

		Success (n = 211)	Failure (n = 7)	*p* Value	OR
Detrusor overactivity		74 (35.07)	3 (42.86)	0.6997	
One hour pad test (g)	<6	188 (89.1)	6 (85.71)	0.5634	
	≧6	23 (10.9)	1 (14.29)		
Q max (mL/s)	<15	126 (59.72)	3 (42.86)	0.4473	
	≧15	85 (40.28)	4 (57.14)		
RU (mL)	<50	82 (38.86)	2 (28.57)	0.7095	
	≧50	129 (61.14)	5 (71.43)		
FS (mL)	<100	57 (27.01)	4 (57.14)	0.0979	
	≧100	154 (72.99)	3 (42.86)		
MCC (mL)	<300	63 (29.86)	2 (28.57)	1	
	≧300	148 (70.14)	5 (71.43)		
Pdet (cm H_2_O)	<25	145 (68.72)	7 (100)	0.103	
	≧25	66 (31.28)	0 (0)		
FUL (mm)	<20	49 (23.22)	5 (71.43)	0.0111	8.27 (1.55, 43.94)
	≧20	162 (76.78)	2 (28.57)		
MUCP (cm H_2_O)	<40	99 (46.92)	2 (28.57)	0.4545	
	≧40	112 (53.08)	5 (71.43)		
OABSS	<6	117 (55.45)	4 (57.14)	1	
	≧6	94 (44.55)	3 (42.86)		
UDI-6	<6	119 (56.4)	3 (42.86)	0.7021	
	≧6	92 (43.6)	4 (57.14)		
IIQ-7	<6	93 (44.08)	4 (57.14)	0.7026	
	≧6	118 (55.92)	3 (42.86)		
ICIQ-SF	<7	135 (63.98)	1 (14.29)		
	≧7	76 (36.02)	6 (85.71)	0.0122	10.66 (1.26, 90.16)
POPDI-6	<10	99 (46.92)	4 (57.14)	0.7097	
	≧10	112 (53.08)	3 (42.86)		
Aa	≦1	97 (45.97)	2 (28.57)	0.4596	
	>1	114 (54.03)	5 (71.43)		
Ba	≦1	34 (16.11)	1 (14.29)	1	
	>1	177 (83.89)	6 (85.71)		
C	≦1	106 (50.24)	3 (42.86)	1	
	>1	105 (49.76)	4 (57.14)		
Ap	≦1	179 (84.83)	6 (85.71)	1	
	>1	32 (15.17)	1 (14.29)		
Bp	≦1	137 (64.93)	4 (57.14)	0.6997	
	>1	74 (35.07)	3 (42.86)		
TVL	≦8	74 (35.07)	4 (57.14)	0.2521	
	>8	137 (64.93)	3 (42.86)		

Data are given as n (%). Q max, maximum flow rate; RU, residual urine; FS, first sensation to void; MCC, maximum cystometric capacity; Pdet, detrusor pressure at peak flow; FUL, functional urethral length; MUCP, maximum urethral closure pressure; OABSS, Overactive Bladder Symptom Scores; UDI-6, Urogenital Distress Inventory; IIQ-7, Incontinence Impact Questionnaire; ICIQ-SF, International Consultation on Incontinence modular Questionnaire–Short Form; POPDI-6, Pelvic Organ Prolapse Distress Inventory 6; TVL, total vaginal length.

## Data Availability

Data are available from the authors upon reasonable request.

## References

[1] Hendrix S.L., Clark A., Nygaard I., Aragaki A., Barnabei V., McTiernan A. (2002). Pelvic organ prolapse in the Women’s Health Initiative: Gravity and gravidity. Am. J. Obstet. Gynecol..

[2] Olsen A.L., Smith V.J., Bergstrom J.O., Colling J.C., Clark A.L. (1997). Epidemiology of surgically managed pelvic organ prolapse and urinary incontinence. Obstet. Gynecol..

[3] Smith F.J., Holman C.D.J., Moorin R.E., Tsokos N. (2010). Lifetime risk of undergoing surgery for pelvic organ prolapse. Obstet. Gynecol..

[4] Jelovsek J.E., Maher C., Barber M.D. (2007). Pelvic organ prolapse. Lancet.

[5] Ng-Stollmann N., Fünfgeld C., Gabriel B., Niesel A. (2020). The international discussion and the new regulations concerning transvaginal mesh implants in pelvic organ prolapse surgery. Int. Urogynecology J..

[6] U.S. Food & Drug Administration (2022). FDA’s Activities: Urogynecologic Surgical Mesh. https://www.fda.gov/medical-devices/urogynecologic-surgical-mesh-implants/fdas-activities-urogynecologic-surgical-mesh.

[7] Zhang C., Sun Z., Yang J., Xu T., Zhu L., Lang J. (2020). Sacrocolpopexy Compared with Transvaginal Mesh Surgery: A Systematic Review and Meta-Analysis. BJOG Int. J. Obstet. Gynaecol..

[8] Bump R.C., Mattiasson A., Bø K., Brubaker L.P., DeLancey J.O., Klarskov P., Shull B.L., Smith A.R. (1996). The standardization of terminology of female pelvic organ prolapse and pelvic floor dysfunction. Am. J. Obs. Gynecol.

[9] Homma Y., Yoshida M., Seki N., Yokoyama O., Kakizaki H., Gotoh M., Yamanishi T., Yamaguchi O., Takeda M., Nishizawa O. (2006). Symptom assessment tool for overactive bladder syndrome—Overactive bladder symptom score. Urology.

[10] Uebersax J.S., Wyman J.F., Shumaker S.A., McClish D.K., Fantl J.A. (1995). Short forms to assess life quality and symptom distress for urinary incontinence in women: The Incontinence Impact Questionnaire and the Urogenital Distress Inventory. Continence Program for Women Research Group. Neurourol. Urodyn..

[11] Ibinaeva I.S., Apolikhina I.A., Makhmedzhanova F.N., Muslimova S.Z. (2012). [ICIQ-SF questionnaire in women with urinary incontinence]. Urologiia.

[12] Barber M.D., Walters M.D., Bump R.C. (2005). Short forms of two condition-specific quality-of-life questionnaires for women with pelvic floor disorders (PFDI-20 and PFIQ-7). Am. J. Obstet. Gynecol..

[13] Ellington D.R., Richter H.E. (2013). Indications, Contraindications, and Complications of Mesh in Surgical Treatment of Pelvic Organ Prolapse. Clin. Obstet. Gynecol..

[14] Tailor V., Digesu A., Swift S.E. (2021). Update in Transvaginal Grafts: The Role of Lightweight Meshes, Biologics, and Hybrid Grafts in Pelvic Organ Prolapse Surgery. Obs. Gynecol Clin..

[15] Naumann G., Hüsch T., Mörgeli C., Kolterer A., Tunn R. (2020). Mesh-augmented transvaginal repair of recurrent or complex anterior pelvic organ prolapse in accordance with the SCENIHR opinion. Int. Urogynecology J..

[16] Wu P.-C., Wu C.-H., Liu Y., Loo Z., Lin K.-L., Long C.-Y. (2020). The clinical and urodynamic outcomes of single-incision mesh surgery using the Uphold system for the treatment of pelvic organ prolapse. Sci. Rep..

[17] Lo T.-S., Rom E., Harun F., Jhang L.-S., Hsieh W.-C., Lin Y.-H. (2024). Anterior–apical Transvaginal Mesh (Calistar-S) for Treatment of Advanced Urogenital Prolapse: Surgical and Functional Outcomes at 1 Year. Int. Urogynecology J..

[18] Long C.Y., Lo T.-S., Wang C.-L., Wu C.-H., Liu C.-M., Su J.-H. (2012). Risk factors of surgical failure following transvaginal mesh repair for the treatment of pelvic organ prolapse. Eur. J. Obstet. Gynecol. Reprod. Biol..

[19] Chen H.-Y., Yeh L.-S., Chang W.-C., Ho M. (2007). Analysis of risk factors associated with surgical failure of inside-out transobturator vaginal tape for treating urodynamic stress incontinence. Int. Urogynecology J. Pelvic Floor Dysfunct..

[20] Amaye-Obu F.A., Drutz H.P. (1999). Surgical management of recurrent stress urinary incontinence: A 12-year experience. Am. J. Obs. Gynecol..

[21] Ker C.-R., Lin K.-L., Loo Z.-X., Juan Y.-S., Long C.-Y. (2018). Comparison of UpholdTM Vaginal Mesh Procedure with Hysterectomy or Uterine Preservation for the Treatment of Pelvic Organ Prolapse. Sci. Rep..

